# The Effect of the COVID-19 Pandemic on Glycemic Monitoring and Other Processes of Care for Type 2 Diabetes: Protocol for a Retrospective Cohort Study

**DOI:** 10.2196/35971

**Published:** 2022-04-22

**Authors:** Mekha Mathew, Jeremy van Vlymen, Bernardo Meza-Torres, William Hinton, Gayathri Delanerolle, Ivelina Yonova, Michael Feher, Xuejuan Fan, Harshana Liyanage, Mark Joy, Fabrizio Carinci, Simon de Lusignan

**Affiliations:** 1 Nuffield Department of Primary Care Health Sciences University of Oxford Oxford United Kingdom; 2 Department of Clinical and Experimental Medicine University of Surrey Guildford United Kingdom; 3 Field Service UK Health Security Agency Birmingham United Kingdom; 4 Department of Statistical Sciences University of Bologna Bologna Italy; 5 Royal College of General Practitioners Research and Surveillance Centre London United Kingdom

**Keywords:** cohort studies, COVID-19, computerized medical record systems, primary health care, type 2 diabetes mellitus, diabetes, glycemic control, monitoring

## Abstract

**Background:**

Social distancing and other nonpharmaceutical interventions to reduce the spread of COVID-19 infection in the United Kingdom have led to substantial changes in delivering ongoing care for patients with chronic conditions, including type 2 diabetes mellitus (T2DM). Clinical guidelines for the management and prevention of complications for people with T2DM delivered in primary care services advise routine annual reviews and were developed when face-to-face consultations were the norm. The shift in consultations from face-to-face to remote consultations caused a reduction in direct clinical contact and may impact the process of care for people with T2DM.

**Objective:**

The aim of this study is to explore the impact of the COVID-19 pandemic’s first year on the monitoring of people with T2DM using routine annual reviews from a national primary care perspective in England.

**Methods:**

A retrospective cohort study of adults with T2DM will be performed using routinely collected primary care data from the Oxford-Royal College of General Practitioners (RCGP) Research and Surveillance Centre (RSC). We will describe the change in the rate of monitoring of hemoglobin A_1c_ (HbA_1c_) between the first year of the COVID-19 pandemic (2020) and the preceding year (2019). We will also report any change in the eight checks that make up the components of these reviews. The change in HbA_1c_ monitoring rates will be determined using a multilevel logistic regression model, adjusting for patient and practice characteristics, and similarly, the change in a composite measure of the completeness of all eight checks will be modeled using ordinal regression. The models will be adjusted for the following patient-level variables: age, gender, socioeconomic status, ethnicity, COVID-19 shielding status, duration of diabetes, and comorbidities. The model will also be adjusted for the following practice-level variables: urban versus rural, practice size, Quality and Outcomes Framework achievement, the National Health Service region, and the proportion of face-to-face consultations. Ethical approval was provided by the University of Oxford Medical Sciences Interdivisional Research Ethics Committee (September 2, 2021, reference R77306/RE001).

**Results:**

The analysis of the data extract will include 3.96 million patients with T2DM across 700 practices, which is 6% of the available Oxford-RCGP RSC adult population. The preliminary results will be submitted to a conference under the domain of primary care. The resulting publication will be submitted to a peer-reviewed journal on diabetes and endocrinology.

**Conclusions:**

The COVID-19 pandemic has impacted the delivery of care, but little is known about the process of caring for people with T2DM. This study will report the impact of the COVID-19 pandemic on these processes of care.

**International Registered Report Identifier (IRRID):**

DERR1-10.2196/35971

## Introduction

### Background

The COVID-19 pandemic and recommended social distancing and other nonpharmaceutical interventions have had a substantial impact on primary care services in the United Kingdom [1,2]. Face-to-face consultations were markedly reduced, and primary care appointments decreased by 64.6% and home visits decreased by 62.6% from the week commencing March 9, 2020, coinciding with national policy changes [3]. This was a consequence of lockdown restrictions and changes made by a series of scientific advisory groups to minimize the risk of exposure to COVID-19, which included encouraging telemedicine as the preferred alternative for face-to-face consultations [1,4]. During the initial stages of the pandemic, primary care services reserved face-to-face consultations for priority appointments, while the policy for delivering routine care via telemedicine was adopted [1]. The changes in methods of consultation and interrupted routine care may have adversely affected the management of people with type 2 diabetes mellitus (T2DM) [5].

Previous studies have identified that missed hemoglobin A_1c_ (HbA_1c_) monitoring appointments is associated with higher HbA_1c_ [6,7]. A recent study showed a 40% reduction in HbA_1c_ testing during the first year of the COVID-19 pandemic compared to the preceding year [8]. It is well established that impaired glycemic control is associated with the increased risk of micro- and macrovascular complications [8,9], indicating the benefit to people with T2DM having regular HbA_1c_ monitoring.

In 2014, the National Institute for Health and Care Excellence (NICE) introduced clinical guidelines for its Quality and Outcomes Framework (QOF) indicator menu to encourage regular monitoring and management of diabetes [10,11]. These are known as *routine annual reviews*, which include eight health checks: HbA_1c_, blood pressure, cholesterol, serum creatinine, urine albumin, foot surveillance, BMI, and smoking status [11]. The proposed indicators are based on the best evidence and are implemented to provide high standards of care and improved results for patients.

However, the extent to which interruptions in primary care services (eg, face-to-face appointments) affected the monitoring of people with T2DM in the United Kingdom during the COVID-19 pandemic has yet to be established. This protocol describes our planned methods to explore the impact of the pandemic on the monitoring of people with T2DM in a UK-based setting.

### Aims and Objectives

Our primary objective is to assess the impact of the COVID-19 pandemic on HbA_1c_ monitoring in people with T2DM. As a secondary objective, we will explore changes in the rates of *routine annual reviews* between the pre–COVID-19 pandemic period and during the first year of the COVID-19 pandemic.

## Methods

### Study Design

We will conduct a retrospective cohort analysis using observational data of adults with T2DM from the Oxford-Royal College of General Practitioners (RCGP) Research and Surveillance Centre (RSC) sentinel network database. The study cohort will be observed at two time points: the year preceding the COVID-19 pandemic (January 1 to December 31, 2019) and the first year of the COVID-19 pandemic (January 1 to December 31, 2020).

### Data Source

The Oxford-RCGP RSC is a sentinel network of volunteer primary care practices across England and Wales, currently comprising more than 15 million patients registered with over 1800 affiliated practices [12]. Pseudonymized coded clinical practice data is uploaded and available in near real time within a secure network, supporting the RSC’s influenza surveillance, identification of epidemics, and other research activity. The network provides a broadly representative sample of the national population [13].

UK primary care data is registration based (ie, patients have unique identifiers—National Health Service [NHS] numbers). Patient electronic health care records are coded using the Systematized Nomenclature of Medicine Clinical Terms (SNOMED CT) code system, a machine-readable clinical vocabulary offering a high degree of granularity and linkage to other classifications and international terminologies [14]. Most of the T2DM management occurs in primary care and pay-for-performance targets to incentivize chronic disease management including T2DM, resulting in well-maintained disease registries, thus ensuring high-quality data for this study [5,15,16].

### Study Population

We will identify adults (aged ≥18 years) with T2DM using diagnosis codes. The cohort for this study will comprise individuals diagnosed with T2DM on or before December 31, 2018, and who are registered with an Oxford-RCGP RSC practice on this date.

### Exposure

The exposure variable will be binary to indicate the first calendar year of the COVID-19 pandemic (January 1 to December 31, 2020) and the year before the pandemic (January 1 to December 31, 2019).

### Outcomes

Our primary outcome measure will be the rate of HbA_1c_ monitoring in the year 2020; this will be compared to HbA_1c_ monitoring in the preceding year.

The secondary outcome will be a measure of the NICE eight health checks that make up the *routine annual review* in each study period. We will sum the number of types of checks conducted in the year per patient and code this to an ordinal variable (≤5 care processes, 6-7 care processes, 8 care processes).

### Study Variables

The study variables of interest are divided into personal characteristics and practice characteristics.

#### Personal Characteristics

The following personal characteristics will be used: age (treated as a continuous variable), gender (male or female), socioeconomic status (quintiles of the Index of Multiple Deprivation [IMD]) according to the national distribution of IMD scores based on the postal code of the patient [17], ethnicity (categorized into major ethnic groups, defined by the Office of National Statistics, Asian, Black, Mixed, White, or other ethnic group) [18,19], COVID-19 shielding status, duration of diabetes, and presence of comorbidities (eg, hypertension and chronic kidney disease; determined by diagnosis codes).

#### Practice Characteristics

For the practice characteristics, urban versus rural primary care practices will be identified from the practice Lower Layer Super Output Area. The practice size, QOF linkage, NHS region (East of England, London, Midlands, North East and Yorkshire, North West, South East, South West), and the number/type of consultations will be taken into account [20].

### Statistical Analysis

The summary statistics will be reported as counts and percentages for categorical data and means (with SDs) for continuous data.

If the missing data is <5% (as routine primary care data is incomplete, we anticipate a small degree of missing data in most, if not all, covariates), no attempt will be made to impute the missing values. Missing data >5% will be handled through multiple imputation by chained equations using the MICE package, version 3.14.0 [21].

To assess the impact of the COVID-19 pandemic on HbA_1c_ monitoring, we will estimate the odds ratio of HbA_1c_ monitoring during the pandemic period and the pre–COVID-19 pandemic period in a multilevel logistic regression model with the first COVID-19 year as an indicator variable. The random intercept model will enable the variation of the impact of the pandemic at the patient, GP, and geographical level to be assessed and enable the estimation of robust effect sizes. We will use ancillary analyses to estimate the population-level effects of covariates measured at the patient and practice level to better describe the impact of interpractice variation.

The secondary outcome, measuring the degree to which patients received all eight *routine annual review* checks, will be modeled using a mixed effects ordinal regression, adding random effects at the practice level. Current research has shown variation in the attainment of the individual checks. We will describe the attainment of the individual checks and achievement of all eight checks. We will adopt the methods used by Holman et al [22] and define the secondary outcome measure as an aggregate score of the varying degrees of partial attainment with an explicit natural ordering. This secondary outcome measure will then be modeled using mixed effects ordinal regression adding random effects at the practice level, accounting for the ranking of the levels of attainment.

The data analysis will be carried out using the statistical software, R version 4.1.1 (The R Foundation for Statistical Computing) [23].

### Ethical Considerations

Research ethics approval (Reference R77306/RE001) was obtained from the University of Oxford Medical Sciences Interdivisional Research Ethics Committee in September 2021. Data are pseudonymized at the point of data extraction and will be held on a secure network at the University of Oxford. This network is compliant with NHS Digital Data Security and Protection toolkit standards [24]. The data analysis will begin in November 2021.

## Results

A power calculation has been made, based on a *Z* test, for the study. A study with an effect size of 0.05 (1% change in monitoring rates) and at a power of 75% will require a total sample size of 237,026 people with T2DM. The power calculation was carried out using G*Power 3.1.9.7 (Buchner A).

The analysis of the data extracted will include 3.96 million patients with T2DM across 700 practices, which is 6% of the available Oxford-RCGP RSC adult population. The preliminary results will be submitted for presentation at a primary care–themed conference. The resulting publication will be submitted for publication in a peer-reviewed journal.

## Discussion

### Overview

This protocol describes how we will explore the effect of the COVID-19 pandemic on the monitoring of people with T2DM by sociodemographics and other individual clinical characteristics. The Oxford-RCGP RSC database is appropriate to use, as the majority of the people with T2DM are managed in primary care.

It is valuable to study primary care practices with respect to diabetes monitoring during the pandemic using evidence-based research. People with T2DM require regular monitoring to minimize the risk of diabetes-associated complications. However, changes in the delivery of primary care services as a result of the COVID-19 pandemic has brought challenges in T2DM assessment and monitoring [2]. The existing literature has focused on an unprecedented reorganization of UK primary care during the pandemic [3]. Remote monitoring systems proved to be feasible and were supported by the current clinical guidelines [3]. However, the study results might represent a considerable burden of unmet need, validating the results of other studies [2,8].

### Strengths and Limitations

The Oxford-RCGP RSC is a large network of primary care practices with wide coverage. Although the network covers England and Wales, previous literature has reported that it provides a representative sample of the UK population, and hence, the final results will be broadly generalizable to the United Kingdom as a whole [13]. Furthermore, the quality of computerized medical records is high due to pay-for-performance targets [15].

However, there are several limitations. Being routinely collected data, there may be issues of missingness and inaccurate recordings. This will be accounted for by using multiple imputation. Moreover, since this is an observational study, one limitation will be unmeasured confounding factors that may result in biased effect estimates, which we will mitigate by performing a sensitivity analysis. Additionally, the enrollment of practices depends on the types of ongoing projects and clinical trials; therefore, our identification of practices will vary. They are signed up to the Oxford-RCGP RSC network on a voluntary basis, which may cause a higher representation of the more affluent areas compared to the average national population [13]. Any additional strengths and limitations observed during the study will be reported in the final manuscript.

### Conclusion

This study will provide insight into the impact of the pandemic in the monitoring of NICE *routine annual reviews* of people with T2DM managed in an English primary care setting. We expect the outcomes from this study to highlight the need for “catch up” in order for primary care to enhance best practices and prevent T2DM complications.

## Abbreviations

HbA_1c_: hemoglobin A_1c_
IMD: Index of Multiple Deprivation
NHS: National Health Service
NICE: National Institute for Health and Care Excellence
QOF: Quality and Outcomes Framework
RCGP: Royal College of General Practitioners
RSC: Research and Surveillance Centre
SNOMED CT: Systematized Nomenclature of Medicine Clinical Terms
T2DM: type 2 diabetes mellitus
